# Pioneer of Biomedical Optics: Brian C. Wilson

**DOI:** 10.1117/1.JBO.30.S3.S34101

**Published:** 2025-10-03

**Authors:** Brian W. Pogue, Lothar Lilge, Stefan Andersson-Engels, Steen J. Madsen, Malini C. Olivo, Alex Vitkin

**Affiliations:** a Thayer School of Engineering at Dartmouth, Hanover, New Hampshire, United States; b University of Toronto, Faculty of Medicine, Department of Medical Biophysics, Toronto, Canada; c University of Johannesburg, Laser Research Centre, Faculty of Health Sciences, Johannesburg, South Africa; d University Health Network, Princess Margaret Cancer Centre, Toronto, Canada; e Tyndall National Institute, Biophotonics@Tyndall, Cork, Ireland; f University College Cork, School of Physics, Cork, Ireland; g University of Nevada, Las Vegas, Department of Health Physics and Diagnostic Sciences, Las Vegas, Nevada, United States; h Agency for Science, Technology and Research (A*STAR), Translational Biophotonics Lab @A*SRL, Singapore

## Abstract

The editorial introduces the special issue honoring Brian C. Wilson as a pioneer of biomedical optics.

## 1 Introduction

Dr Brian Wilson’s career is foundational in biomedical optics and biophotonics. He invented and articulated many key aspects of enabling technologies, deepened our fundamental theoretical understanding, and drove the medical translation of novel devices. His impact could be quantified in many ways—traditional measures such as publications, funded grants, citations, trainees, etc. are indeed all outstanding—but perhaps his larger and longer-lasting impact has been his altruistic approach that infects his colleagues with scientific curiosity, thereby initiating the establishment of large research group in biophotonics and advancing collaborations across institutions, countries and disciplines. The overarching goal of clinical impact, advancing hand-in-hand with the underlying approaches of basic scientific insight and technological innovation, is his signature approach. This unique blend of altruism in interactions, innovation, and scientific rigor in methodology has yielded widespread networks of collaborators across all continents, and justly earned Brian the reputation of being one of the most desired biophotonics consultants to major institutions and companies across the world. His role in the application of optics in cancer research also positioned him at the forefront of many advisory boards, both national and international, that shape the science of optical devices to enhance and advance human health.

**Fig. 1:**
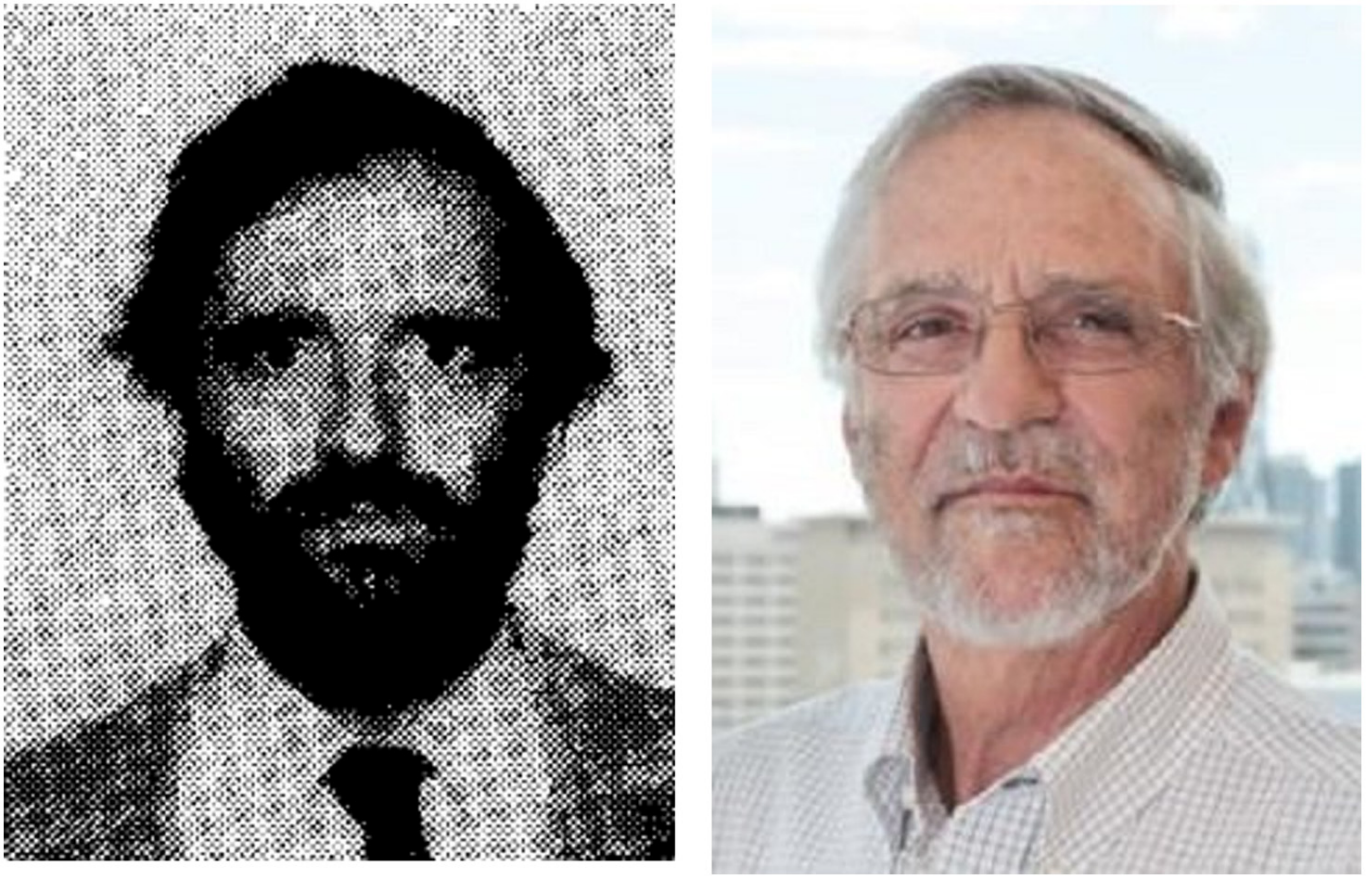
An early pre-digital photograph of Brian C. Wilson associated with a paper in the IEEE (bearing a striking resemblance to a young Ringo Starr as relayed by Dr. Michael Patterson) and a more recent digital photo from Toronto.

**Fig. 2:**
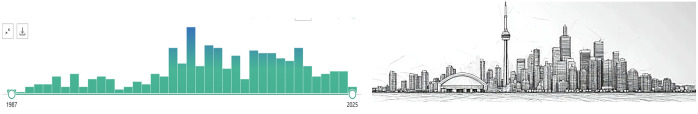
Brian C. Wilson publication record showing (a) continuous progression over nearly 4 decades, and (b) a surprising similarity to the Toronto skyline.

**Fig. 3:**
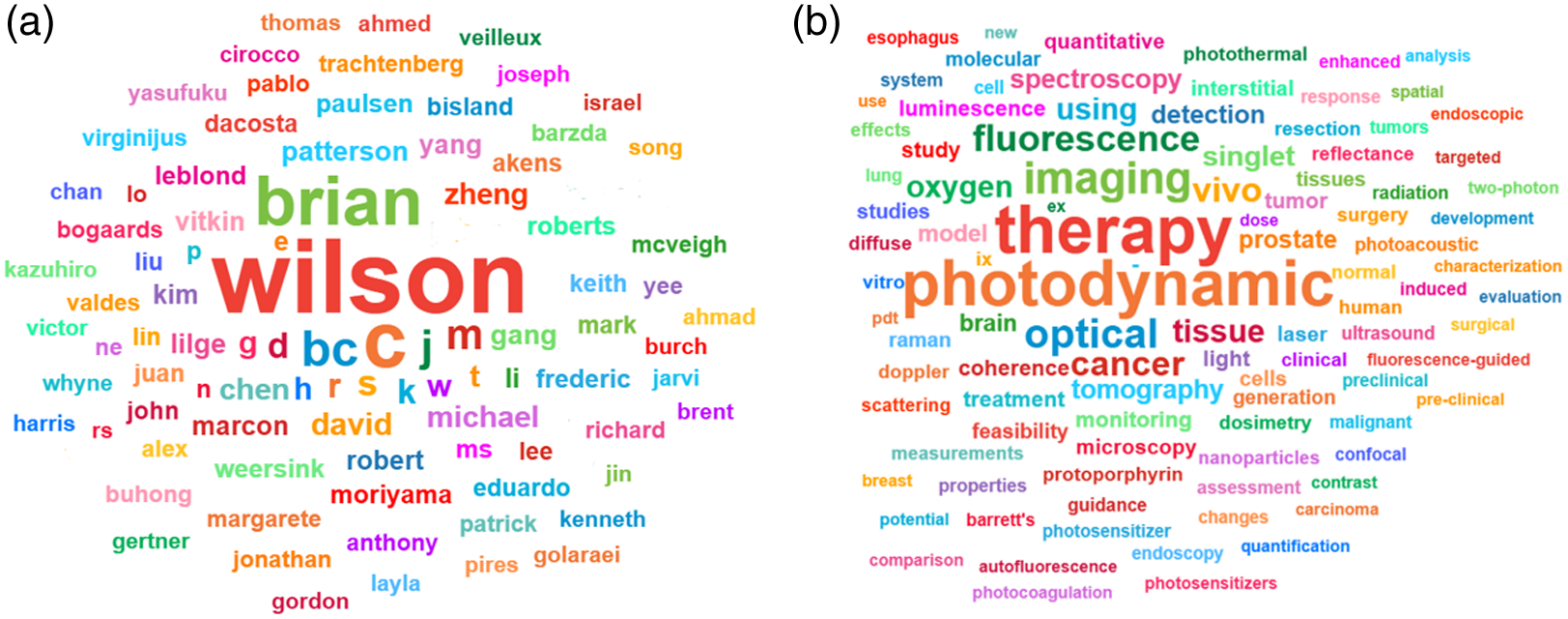
Wordle of key publication co-authors (a) and key scientific words from his publications (b) over the course of 45+ years of work.

Brian’s scientific journey, and the subsequent expansion of biophotonics R&D in Canada, occurred in two major steps, first when he launched the original Biomedical Optics Group at the Hamilton Regional Cancer Centre/McMaster University [Fig. 1(a)] and again when he established the *Laboratory for Applied Biophotonics* at Princess Margaret Hospital/University of Toronto [Fig. 1(b)]. He linked this with Photonics Research Ontario, launching a unique industry User facility, and with the Canadian Institute for Photonics Innovation. Collaborations with photonics groups across multiple university departments were initiated, and his impact expanded across the entire field of biomedical optics. His publication list over four decades is extensive (Fig. 2) and spans a wide range of topics, with a lengthy list of collaborators (Fig. 3).

Dr. Wilson completed his PhD in experimental particle physics University of Glasgow and CERN, Geneva. He subsequently transitioned to medical physics at the Royal Marsden Hospital/Institute of Cancer Research in London, where he worked on therapeutic and diagnostic radiology/nuclear medicine. This continued upon moving to Australia, and then in Canada, where he developed a parallel career in biophotonics, particularly applied to cancer research diagnostics and therapeutics.

His impact in the field is broader than his specific scientific contributions and extends internationally. His infectious spirit and genuine collegiality have inspired many others and provided innovative ideas to other institutions, building their own biophotonics initiatives. Examples of prominent collaborators are illustrated in Fig. 3.

## 2 Founding Principles of Photodynamic Therapy – Initiating Canadian Biomedical Optics

**Fig. 4:**
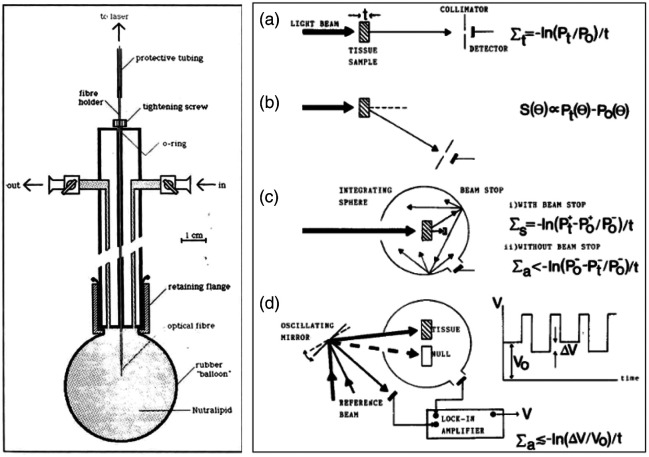
(a) Balloon irradiator devised to illuminate PDT in the resection bed of the brain during neurosurgical treatment, used in many clinical cases.^7^ This approach was used in several other intracavitary irradiation PDT trials. (b) Measurement of tissue optical properties was pioneered using diffuse transmission and reflectance.^5^ This early work stimulated a large field of tissue optics research that blossomed throughout the 1990s.

**Fig. 5:**
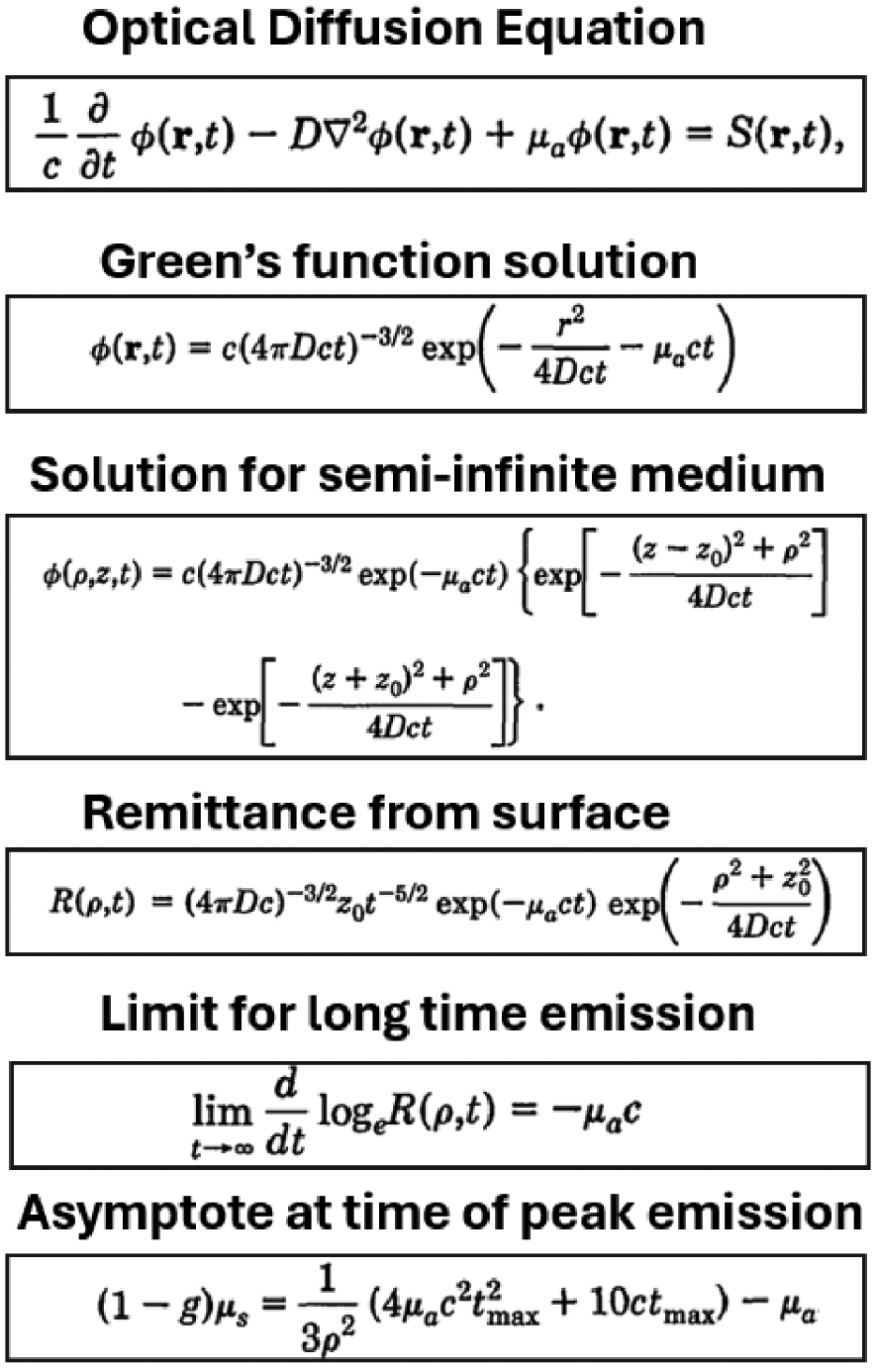
The time-dependent diffusion equation was first solved analytically by Michael Patterson, Britton Chance, and Brian Wilson,^11^ leading to practical solutions that separate absorption from scattering in the measured data sets, in the latter two equations.

His transition to biomedical optics occurred when he stumbled across a seminal paper on problems in dosimetry of the emerging modality of photodynamic therapy, PDT. This led to fundamental studies of tissue optics, starting with the first paper on the use of the Monte Carlo model of light propagation in tissue,^1^ followed by experimental techniques for measuring tissue optical properties^2^^–^^5^ and analytic modeling.^5^^,^^6^ The first clinical application was in brain tumors, in collaboration with Toronto neurosurgeon Paul Muller,^3^^,^^7^^–^^9^ culminating in a National Institutes of Health (NIH) sponsored Phase III clinical trial: his first intracavity balloon irradiator for PDT is shown in Fig. 4(a), and systems for light-tissue interaction measurements in Fig. 4(b). This initial work led to foundational studies with Michael S. Patterson on light-tissue modeling through radiation transport/diffusion theory,^10^^–^^13^ illustrated in Fig. 5. As described by Dr. Patterson, “Starting from literally nothing, he secured three peer-reviewed grants: one dealt with PDT dosimetry and light propagation in tissue, another focused on radiolabelled photosensitizers for PET imaging produced at the McMaster nuclear reactor, and the third was a clinical trial of intraoperative PDT for brain tumors with Paul Muller, a neurosurgeon in Toronto.” From there things continued to snowball bringing in other investigators in medical physics and other aspects of the Hamilton Regional Cancer Center as well as McMaster University, and a stream of graduate students and postdoctoral researchers.

A seminal theoretical paper by Patterson, Wilson and Chance in 1989^11^ on time-resolved light propagation in tissue (cited > 3000 times) shaped research worldwide and helped lay the theoretical foundations of applications such as functional near-infrared spectroscopy (fNIRS) and diffuse optical tomography (DOT) as well as many other areas requiring quantitative separation of the effects of absorption and transport scattering coefficients. Several methods and devices for tissue spectroscopy utilizing spatially resolved light measurements were also developed.^14^^,^^15^

In parallel, Dr. Wilson carried out fundamental photobiological studies on PDT-induced resistance with colleague Gurmit Singh,^16^^–^^18^ on fluence-rate effects, and methods for quantitative PDT dosimetry,^19^^–^^21^ as well as establishing the fundamental concept of PDT threshold dose^22^^–^^25^ that is used widely in preclinical and clinical PDT applications.

## 3 Explosion of Biophotonics – Toronto Years

His move to Toronto in 1993 as Head of the Division of Clinical Physics at the Princess Margaret Hospital and Professor of Medical Biophysics at the University of Toronto enabled him to expand his research in new directions, including advanced instrumentation for singlet oxygen dosimetry,^26^^–^^39^ Doppler and speckle variance OCT angiography,^40^^–^^46^ photobleaching^36^^,^^47^^,^^48^ and 2-photon PDT.^49^^–^^54^ A new era in PDT dosimetry began in the 1990s with a seminal concept paper, in which he coalesced several concepts into the paradigm of implicit dosimetry.^55^

He also recognized the potential of PDT in non-cancer applications, such as targeting bacteria and viruses, including for organ transplantation and, most recently, SARS-CoV-2, which included a first-in-class clinical trial for nasal photo-decontamination.^54^^,^^56^ His broad perspective on the strengths and opportunities in PDT has helped to continue its successful implementation in a range of clinical trials.^57^

A major spin-off from his PDT work was fluorescence spectroscopy/imaging-based diagnostics, in which he led many early conceptual and technological studies, clinical trials, and commercial translation, particularly based on endogenous and exogenous fluorophores. Notable here is his collaboration with Toronto GI endoscopist Dr. Norman Marcon on the detection of dysplastic lesions in Barrett’s esophagus and adenomatous polyps in the colon.^58^^–^^60^

**Fig. 6:**
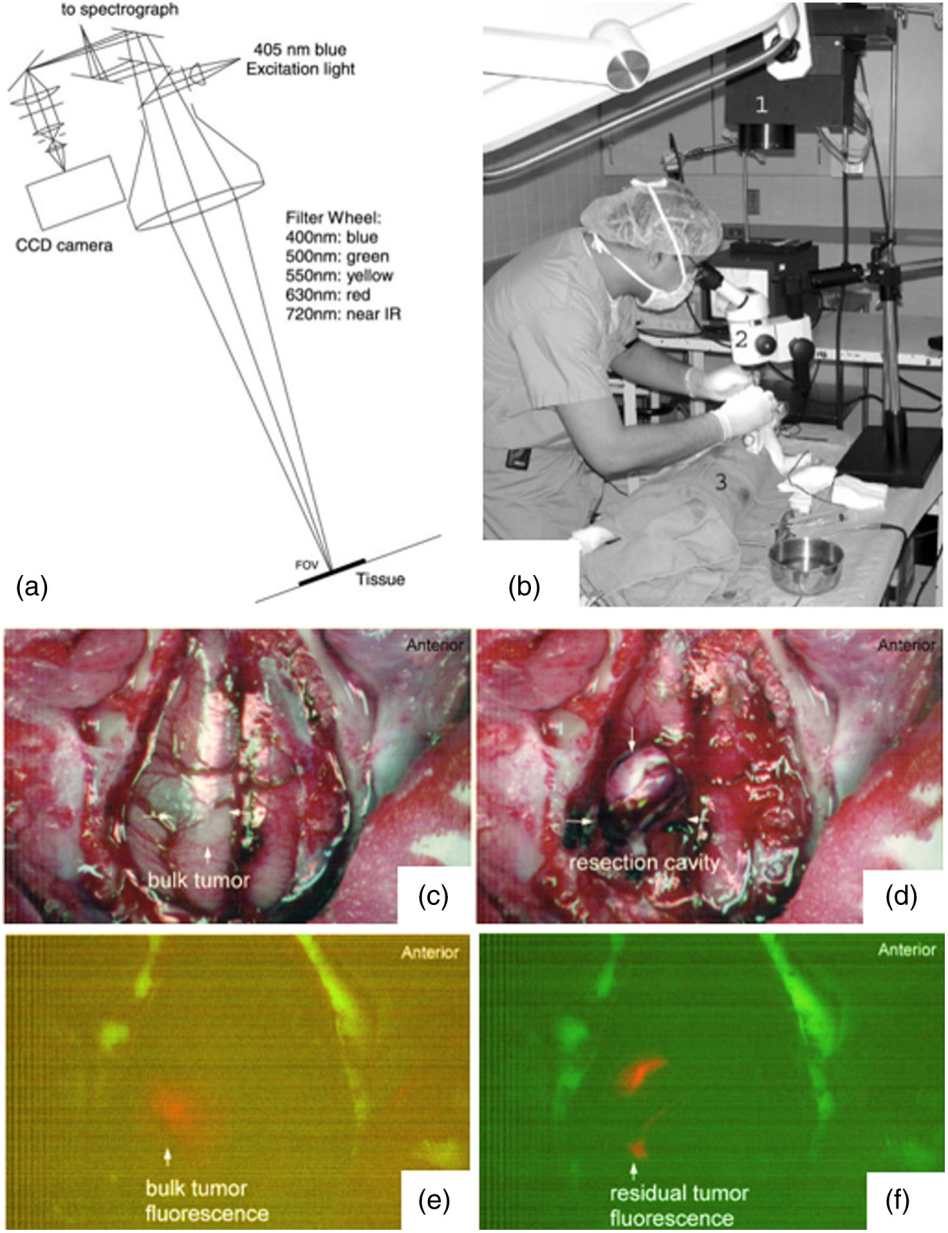
(a) Design of an early co-axial multi-wavelength fluorescence imaging and spectroscopy system. (b) Surgical setup with the prototype system and examples of white light before resection (c), after resection (d) and corresponding fluorescence images (e) and (f) in intracranial rabbit tumor model.^62^

Broad interest in fluorescence diagnostics merged with his focus on PDT in glioma tumors, developing an extensive series of studies with collaborators, using Photofrin fluorescence for surgical guidance (FGS) with graduate student Victor Yang and others.^61^ This was at the onset of the ALA-PpIX fluorescence-guided tumor resection, now adopted throughout much of the neurosurgery community. In Toronto, Dr. Wilson led several key scientific and technical developments for ALA-PpIX based FGS, as illustrated in Fig. 6,^62^^–^^65^ mainly focused on quantitative methods^66^ and registration systems that preceded the clinical adoption of FGS in 2007 in Germany^67^ and 2017 in the USA.^68^^,^^69^ This theme included a long collaboration with biomedical engineer Dr. Keith Paulsen and neurosurgeon David Roberts MD, at Dartmouth-Hitchcock Medical Center, including launching a company and publishing multiple technical advances and clinical trials.^70^^–^^78^ Over the course of several decades, he also worked with collaborators on 2-photon microscopy for cancer pathology,^49^^–^^53^^,^^79^ as well as Raman spectral diagnostics.^60^^,^^80^^–^^84^

These advances were accompanied by interest in contrast agents and in the 2000s the push in nanotechnology for multimodal imaging and theranostics. This spans C-60 (Bucky balls), quantum dots, SERS-coated gold nanoparticles, and scintillation nanoparticles for x-ray-mediated PDT.^85^^–^^87^ A major focus for the past decade has been with multifunctional porphyrin-lipid nanoparticles (porphysomes) in collaboration with chemist Dr. Gang Zheng, whom Dr. Wilson recruited to Toronto.^88^^–^^93^

A unique aspect of his career is his increasing focus on photobiology in addition to optical biophysics and engineering. Most recently, this has included functional and mechanistic studies of 2-photon activation and immune stimulation in photodynamic therapy, applied to cutaneous and ocular melanoma.^54^^,^^94^^,^^95^ Characteristically, this foundational work is in collaboration with Dr. Layla Pires, first as a graduate student and postdoctoral fellow co-supervised in Toronto and Brazil (with Drs. Cristina Kurachi and Vanderlei Bagnato), and now a faculty member at Texas A&M University.

## 4 Shaping Careers, Institutions, and the International Landscape in Biomedical Optics and Biophotonics

**Fig. 7:**
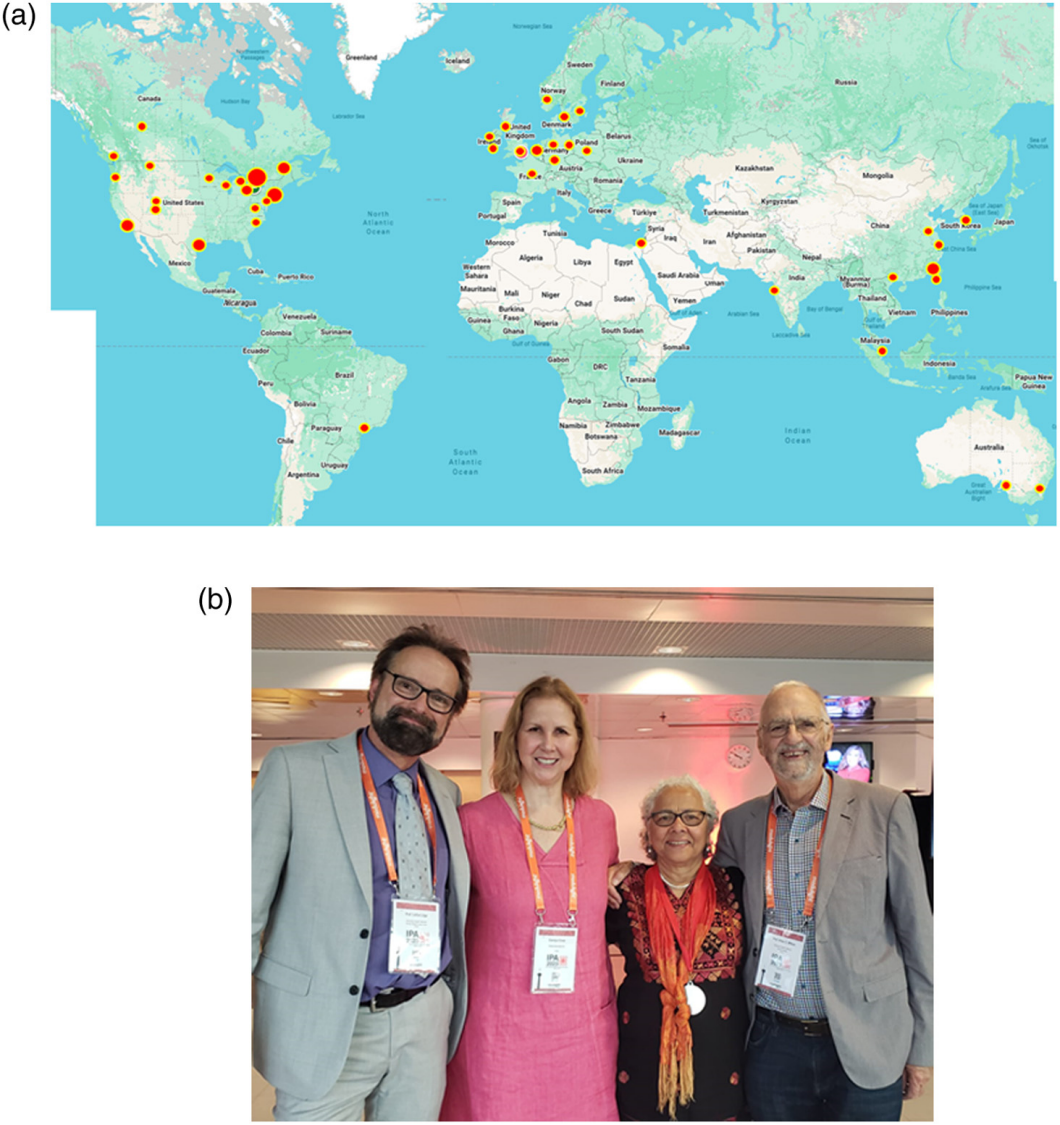
(a) The locations of co-author institutions for individuals who published multiple papers with Prof. Wilson illustrate his impact throughout extensive global collaborations, especially with former trainees. (b) At a recent International Photodynamic Society Meeting in Finland, with Lothar Lilge (UHN/U Toronto), Carolyn Cross (Ondine Biomed Inc), and Tayyaba Hasan (MGH/Harvard).

To date Brian has supervised 20 MS students, 25 PhD/MD-PhD students, and 41 postdoctoral trainees (including Clinical Fellows), with another 8 trainees currently. The number of senior faculty who owe their career advancement to formal and informal mentorship and collaboration with Dr. Wilson is in the hundreds. Appreciation of his mentorship from his own trainees and those of his collaborators is evident in the fact that he is continues to publish with many of them. A key part of his approach to science has been cross-institutional collaborations, focusing on advancing the field rather than simply advancing his own program or institution. This is evident in the numerous advisory boards and visiting appointments he has maintained over the past 4+ decades. Importantly, he has been advisory to foundational centers on biophotonics at the Wellman Center for Photomedicine in Boston^96^ (where he was visiting professor for many years in the 1980s and 90s); the Beckman Laser Institute in Irvine, California; the Australian Research Center in Nanoscale Biophotonics in Adelaide; Tyndall National Institute for Photonics in Cork, Ireland; UC Davis Biophotonics Center, California, Dartmouth College Cancer Center, New Hampshire, University of Sao Paulo-Sao Carlos, Brazil; and the School of Photonics & EE at Fujian Normal University, China. He has been on nearly every cancer research advisory group within Canada at one time or another throughout his career, and many photonics industry advisory boards. He has served on grant review panels and site visits throughout the world, extensively with the US NIH, National Science Foundation, US-Army, and the US MFEL Program. His role in cancer research and treatment, as well as biophysics, can be seen in the extensive growth of biophotonics research and translation within the greater University of Toronto system, throughout Ontario and Canada, and well beyond national borders, illustrated in Fig. 7. A Scopus search indicates that he has over 1200 unique co-authors on his publications, indicating a truly unparalleled level of collaboration.

**Fig. 8:**
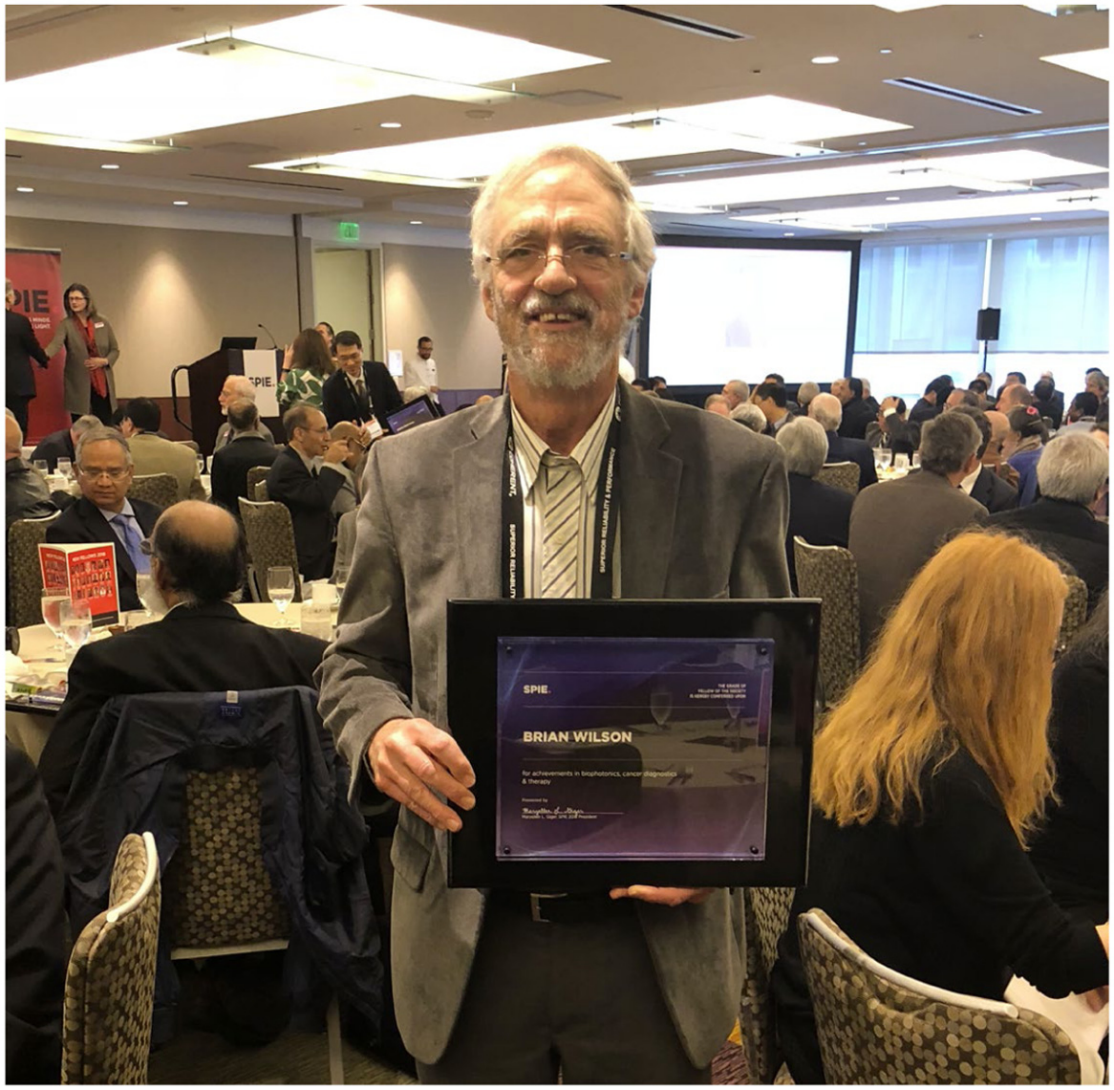
Receiving SPIE Fellow status in 2018, 4 years after receiving the SPIE Britton Chance Award.

His positions in international advisory boards have gone on to shape biophotonics worldwide. His practical approach to translating research findings for human benefit naturally led him to become involved with companies as a founder or advisory board member in over 23 ventures. This contribution continues and will help shape the face of clinical and commercial biophotonics in the future.

The number of conferences he organized over his career is extensive, and his involvement continues to this day. He has been a committee chair of nearly every biomedical optics and photodynamic therapy conference that has existed. This includes a NATO Advanced Institute on Biophotonics, the Gordon Research Conferences, Engineering Conferences International, the International Society for Optics Within Lifesciences (OWLS), Photonics North, and the International Photodynamic Association, to name a few. He initiated and presided over Biophotonics Week in Quebec in 2010, which comprised five separate complementary conferences in biophotonics. He has actively participated in all the biomedical optics major conferences for decades, leading where needed, such as SPIE-BiOS and Optica Biomed. His real forte was shaping focused topical conferences with key speakers and leading groups to advance fields rather than just report on results.

Among his notable awards are the Mark Award of the American Society for Laser Medicine & Surgery, a Lifetime Achievement Award from the NIH Workshop on Optical Imaging, the Robert L. Noble Prize in Cancer Research, the OSA/Optica Michael S. Feld Biophotonics Award, the SPIE Britton Chance Award (Fig. 8), and the Richard Hill Mentorship Award from Princess Margaret Cancer Center.

Dr. Wilson continues his career unabated, carrying on research, advising, and consulting at a level unheard of for most academics. He is truly a foundational pioneer of biomedical optics/biophotonics and translational cancer research, with immense local, regional, and global impact.
